# 2140: Estimating microscopic structures of glomeruli in renal pathology

**DOI:** 10.1017/cts.2017.58

**Published:** 2018-05-10

**Authors:** Pinaki Sarder, Rabi Yacoub, John E. Tomaszewski

**Affiliations:** University at Buffalo, State University of New York, Buffalo, NY, USA

## Abstract

OBJECTIVES/SPECIFIC AIMS: (i) Digitally quantify pathologically relevant glomerular microcompartmental structures in murine renal tissue histopathology images. (ii) Digitally model disease trajectory in a mouse model of diabetic nephropathy (DN). METHODS/STUDY POPULATION: We have developed a computational pipeline for glomerular structural compartmentalization based on Gabor filtering and multiresolution community detection (MCD). The MCD method employs improved, efficient optimization of a Potts model Hamiltonian, adopted from theoretical physics, modeling interacting electron spins. The method is parameter-free and capable of simultaneously selecting relevant structure at all biologically relevant scales. It can segment glomerular compartments from a large image containing hundreds of glomeruli in seconds for quantification—which is not possible manually. We will analyze the performance of our computational pipeline in healthy and streptozotocin induced DN mice using renal tissue images, and model the structural distributions of automatically quantified glomerular features as a function of DN progression. The performance of this structural-disease model will be compared with existing visual quantification methods used by pathologists in the clinic. RESULTS/ANTICIPATED RESULTS: Computational modeling will reveal digital biomarkers for early proteinuria in DN, able to predict disease trajectory with greater precision and accuracy than manual inspection alone. DISCUSSION/SIGNIFICANCE OF IMPACT: Automated detection of microscopic structural changes in renal tissue will eventually lead to objective, standardized diagnosis, reflecting cost savings for DN through discovery of digital biomarkers hidden within numerical structural distributions. This computational study will pave the path for the creation of new digital tools which provide clinicians invaluable quantitative information about expected patient disease trajectory, enabling earlier clinical predictions and development of early therapeutic interventions for kidney diseases.

